# Dataset for reservoir impoundment operation coupling parallel dynamic programming with importance sampling and successive approximation

**DOI:** 10.1016/j.dib.2019.104440

**Published:** 2019-08-28

**Authors:** Shaokun He, Shenglian Guo, Kebing Chen, Lele Deng, Zhen Liao, Feng Xiong, Jiabo Yin

**Affiliations:** State Key Laboratory of Water Resources and Hydropower Engineering Science, Wuhan University, 430072, China

**Keywords:** Cascade reservoirs, Impoundment operation, Yangtze river basin, Streamflow observation, Parallel dynamic programming, Importance sampling, Seasonal top of buffer pools

## Abstract

The dataset contains reservoir characteristic parameters, streamflow series of reservoirs in the upper Yangtze River, the standard operating rules (SORs) and the seasonal top of buffer pools (seasonal TBPs) for these reservoirs, which were provided by the Yangtze River Commission. Moreover, annual hydropower of these reservoirs is tested to evaluate operation performance. These research materials are related to the research article in *Advances in Water Resources*, entitled ‘Optimal impoundment operation for cascade reservoirs coupling parallel dynamic programming with importance sampling and successive approximation’ (He et al., 2019). The dataset could be used to derive optimal operating rules to explore the potential benefits of water resources via our proposed algorithm (importance sampling – parallel dynamic programming, IS-PDP) in different runoff scenarios. It can also be further applied for water resources management and other potential users.

**Table:** Specifications Table

Subject	Environmental Science; Water resources;
Specific subject area	Water Science and Technology; Hydrology and Water Resources
Type of data	Table and figures
How data were acquired	Hydrological measurement Software: MATLAB code (available on https://data.mendeley.com/datasets/6dhxpdh7cy/1)
Data format	Raw, analysed
Parameters for data collection	Reservoir characteristic parameters (e.g., the relationship between forebay water level and reservoir storage, water release and downstream water level and so on); The standard operating policy of reservoirs; The daily inflow series of reservoirs; Seasonal top of buffer pools for these reservoirs; Annual hydropower of these reservoirs.
Description of data collection	Reservoir characteristic parameters are provided by the Yangtze River Commission The streamflow series from Aug. 1st to Oct. 31st spanning over sixty years was observed from the nearby hydrological gauging stations. Seasonal top of buffer pools for these reservoirs can be referred to the official document *National Flood Control and Drought Relief Headquarters Reply on the Optimal Scheduling Program for cascade reservoirs in the Upper Yangtze River* and obtained by the reservoir flood routing approach which is described in some open access literatures [see Reference [2], [3]]. Annual hydropower of these reservoirs are acquired by reservoir operation model.
Data source location	The upper Yangtze River basin (25–35°N latitude, 90–113°E longitude), China
Data accessibility	Mendeley Data, DOI: https://dx.doi.org/10.17632/j3sn5twdz2.2 Direct URL to data: https://data.mendeley.com/datasets/j3sn5twdz2/2
Related research article	Shaokun He, Shenglian Guo, Kebing Chen, Lele Deng, Zhen Liao, Feng Xiong, Jiabo Yin, ‘Optimal impoundment operation for cascade reservoirs coupling parallel dynamic programming with importance sampling and successive approximation’, Advances in Water Resources, 131(2019) 103375 [1].

**Table:** 

**Value of the data** • The dataset can be used to test the performance between computation efficiency and optimization results of different algorithms as Ref. [4] did. • This dataset is beneficial for policy decision makers, water and energy managers, government organization and some research institutes to make in-depth analysis. • This dataset can be used as a seed, and further expansion of the dataset would be extremely valuable. Collecting similar datasets from other reservoirs in different regions will further allow researchers to find the commons and special issues of those reservoirs.

## 1 Data

**Fig. 1:**
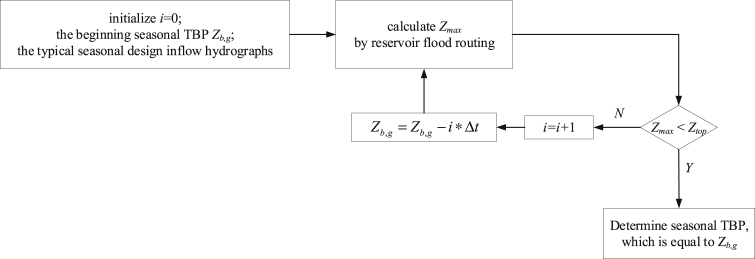
The flowchart of determining seasonal TBP module.

**Fig. 2:**
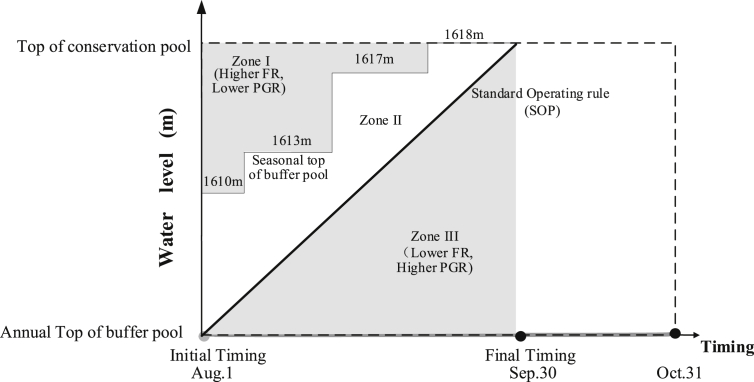
The schematic diagram of the optimization space of Reservoir Li-Yuan (LY), presented in reference [1].

**Fig. 3:**
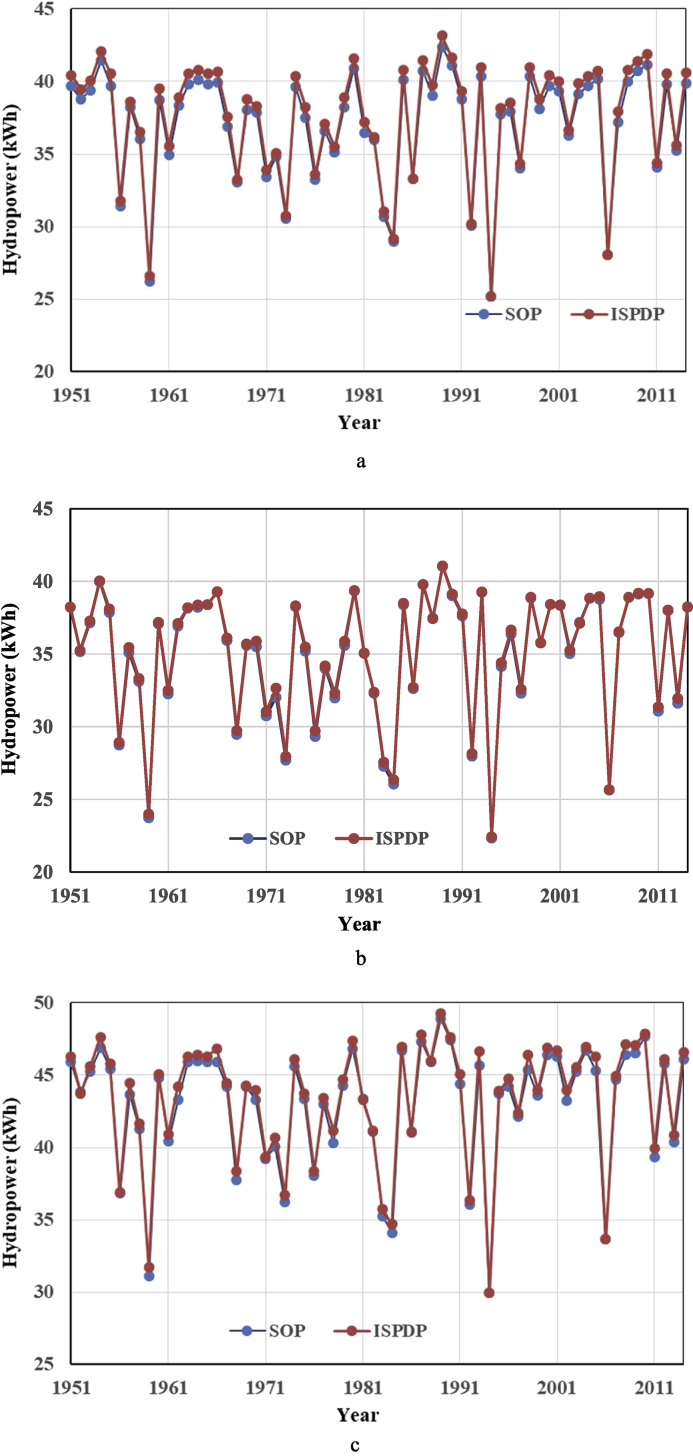
**Annual hydropower of two different rules for LY, AH and JAQ reservoirs**. a Annual hydropower of the LY Reservoir. b Annual hydropower of the LY Reservoir. c Annual hydropower of the JAQ Reservoir.

**Fig. 4:**
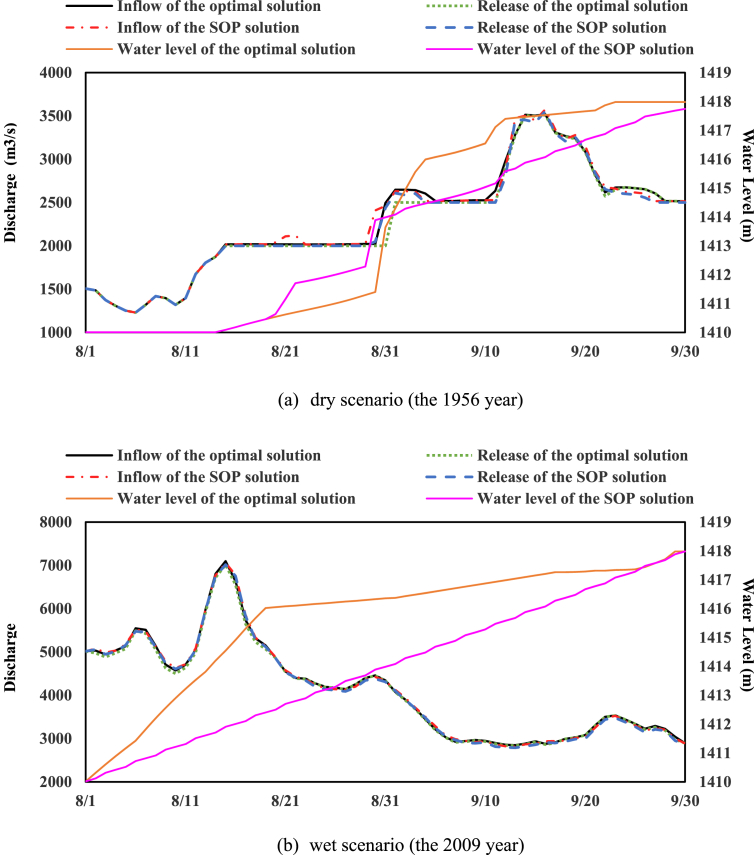
The different impoundment process of two operating policies of JAQ reservoir.

The research dataset [5] of cascade reservoirs located in the upper Yangtze River region contains: 1) daily streamflow series during the impoundment periods (Aug. 1st - Oct. 31st), which were observed in their nearby hydrological gauging stations; 2) basic statistics of the cascade reservoirs, including annual top of buffer pool, top of total conservation pool, installed hydropower capacity and so on, were supplied by the Yangtze River Commission; 3) seasonal top of buffer pool (seasonal TBP), one of the most critical boundary constraints to limit the maximum allowable storage at different stages of the impoundment period, was estimated and determined; 4) hydropower generation was simulated by reservoir operation model. Fig. 1 describes the reservoir flood routing approach [6], [7] in detail to determine seasonal TBP. Fig. 2 describes the optimization space of a reservoir during impoundment period, conventionally, the feasible optimization space consists of seasonal TBP and Standard Operating Rule (SOP). Table 1 then displays the results of seasonal TBP for cascade reservoirs at different periods with reference to *National Flood Control and Drought Relief Headquarters reply on the optimal scheduling program for cascade reservoirs in the upper Yangtze River*. Fig. 3 displays two different operating rule performances via comparing the annual hydropower results. Fig. 4 compare the different impoundment processes of the two operating policies.

**Table 1:** Seasonal top of buffer pool for cascade reservoirs at different periods (Unit: m), presented in reference [1].

Reservoir	seasonal top of buffer pool (seasonal TBP)					
	Aug.15^th^	Aug.25^th^	Sep.1^st^	Sep.10^th^	Sep.30^th^	Oct.31^st^
LY	1610	1613	1617	1617	1618	1618
AH	1497	1500	1503	1503	1504	1504
JAQ	1415	1417	1418	1418	1418	1418

## 2 Experimental design, materials, and methods

Seasonal TBP should be reemphasized here, which defines the maximum reservoir storage at different periods for flood hazards prevention and constrains a reasonable impoundment process. It is determined by *National Flood Control and Drought Relief Headquarters reply on the optimal scheduling program for cascade reservoirs in the upper Yangtze River* and the reservoir flood routing approach simultaneously. The procedure is described as follows:

Step 1: Input initial data series: the initial seasonal TBP (i.e., Z_*b,g*_) which is determined by *National Flood Control and Drought Relief Headquarters reply on the optimal scheduling program for cascade reservoirs in the upper Yangtze River*, and typical seasonal design inflow hydrographs for a given return period, etc.
Step 2: Based on the initial water level *Z*_*b,g*_, calculating the highest water level *Z*_*max*_ by reservoir flood routing according to the operating rules during the impoundment period. If *Z*_*max*_ is less than the top of conservation pool level *Z*_*top*_, seasonal TBP is taken as *Z*_*b,g*_; Otherwise, *Z*_*b,g*_ is decreased by a given step size (ΔZ = 0.1) with the iterative calculation;

Then, the optimization space is structured. Fig. 2 (in Ref. [1]) takes an example of Reservoir Li-Yuan (LY) to draw the schematic diagram of the optimization space (Zone II) for clarity. The season TBPs of LY at time Aug.15th, Aug.25th, Sep.1st, Sep.10th and Sep.30th are 1610m, 1613m, 1617m, 1617m and 1618m, respectively. And the seasonal TBPs of A-hai (AH), Jin-An-Qiao (JAQ) are also inversely related to the start time of impoundment operation (in Table 1). The reason is that the possibility of large flood events decreases over impoundment time, which results in higher seasonal TBP of reservoir to prevent smaller flood event.

Since most datasets can be derived from the authoritative resources (e.g., the basic statistics of the cascade reservoirs are provided by the Yangtze River Commission), there are a few datasets left to be obtained, such as streamflow series of downstream reservoir influenced by upstream reservoir operation, which are calculated by the Muskingum method.

(1) $$Q_{z}\left(t\right)=C_{0}R\left(t\right)+C_{1}R\left(t-1\right)+C_{2}Q_{z}\left(t-1\right)$$

where $Q_{z}\left(t\right)$ and R(t) is the inflow of downstream reservoir and outflow of up streamflow, respectively. $C_{0}$, $C_{1}$ and $C_{2}$ are the channel routing coefficients where the equation $C_{0}+C_{1}+C_{2}=1$ must be satisfied. The water loss from evaporation is not considered here.

All collected datasets can be input to a simulation model optimized by our novel algorithm (Importance sampling – parallel dynamic programming, IS-PDP), the algorithm has further detailed description in Ref. 1. The model is simulated by MATLAB software with a personal computer equipped with 3.30 GHz processor and 16.0 GB RAM as well as up to 16 processors. The algorithm has a centre part of using parallel technique.

The parallelization technique can be realized in the MATLAB environment by structuring the framework of ‘*parfor … end*’. It has an abstract demonstration below:

**Figure:**
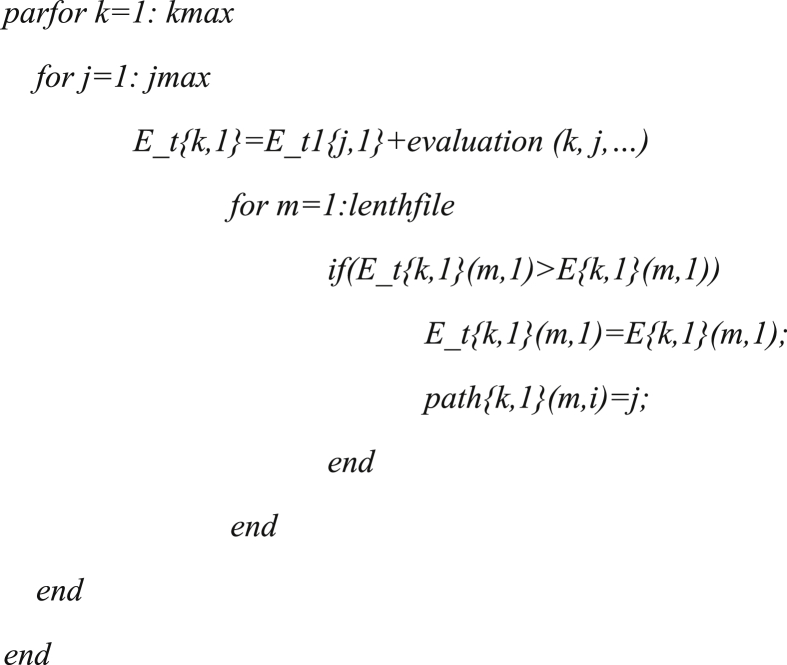

where *kmax* and *jmax* are the number of discrete state combinations at stage *t* and stage *t*-1, respectively; *E_t1{j,1}* is the optimal cumulative return from the initial state at first stage to the *j*th state combination at *t*-1st time; and the function symbol ‘evaluation’ is the objection value at period *t*; lengthfile ‘*m*’ is the years of streamflow series; *path{k,1}(m,i)* records the optimal path; the code can be referred to the website: https://github.com/hsk0059/IS-PDP-and-PDP.

Fig. 3 presents the annual hydropower of two different rules optimized by our proposed IS-PDP and traditional parallel dynamic programming (PDP) [8], respectively.

The annual hydropower obtained by the optimal operating rule has some increase in comparison to SOP. The increase of annual mean hydropower of LY reservoir (installed hydropower capacity: 2.40 GW), AH reservoir (installed hydropower capacity: 2.00 GW) and JAQ (installed hydropower capacity: 2.40 GW) reservoir is 54.14 million, 13.73 million and 41.52 million KWh, respectively. It can been seen that the incremental hydropower generation of reservoirs with larger installed capacity via optimal operation is more than that of reservoirs with smaller installed capacity. In other words, tapping hydropower potential of reservoirs with larger hydropower capacity is more valuable.

Moreover, in order to verify the reasonability of impoundment process, Fig. 4 takes JAQ reservoir as an example to compare the different impoundment process of two operating policies, where the 1956 year represents the dry scenario; the 2009 year represents another wet scenario.

In both scenarios, the outflow of two impoundment process have no much difference. However, the optimal rule can guide water level of JAQ reservoir up to the top of conservation pool in 1956 year whereas the SOP failed, the optimal rule can reserve more water storage to generate more hydropower than SOP meanwhile control water level not higher than seasonal TBP. The optimal rule can generate more hydropower in wet scenario, because more abundant water resources in wet year can be utilized.

In summary, the dataset can be verified that the proposed IS-PDP algorithm can behave superior than some common operating policies (e.g., SOP) in terms of some aspects. This dataset also can be further applied for water resources management and other potential users.
